# Modeling transportation of efavirenz: inference on possibility of mixed modes of transportation and kinetic solubility

**DOI:** 10.3389/fphar.2015.00121

**Published:** 2015-06-08

**Authors:** Tafireyi Nemaura

**Affiliations:** ^1^Department of Clinical Pharmacology, University of ZimbabweHarare, Zimbabwe; ^2^Department of Applied Mathematics, National University of Science and TechnologyBulawayo, Zimbabwe

**Keywords:** efavirenz, models, advection rate, passive transportation and energy dependent transportation rate, convection rate, advection rate constant, kinetic solubility

## Abstract

Understanding drug transportation mechanisms in the human body is of paramount importance in modeling Pharmacokinetic-Pharmacodynamic relationships. This work gives a novel general model of efavirenz transportation projections based on concentrations simulated from patients on a dose of 600 mg. The work puts forward a proposition that transportation can wholly be modeled by concentration and time in a uniform volumetric space. Furthermore, movement entities are used to inform the state of “kinetic solubility” of a solution. There is use of Ricker's model, and forms of the Hill's equation in modeling transportation. Characterization on the movement rates of solution particle are suggested in relation to advection rate of solution particle. At turning points on the transportation rate of solution particle vs. concentration curve, a suggestion of possibly change of dominance in the mode of transportation and saturation is made. There are four movement rates postulated at primary micro-level transportation, that are attributed to convection, diffusion [passive transportation (*E_I_*)] and energy dependent system transportation (*E_D_*) in relation to advection. Furthermore, a new parameter is introduced which is defined as an advection rate constant of solution particle. It is postulated to be dependent on two rate constants of solution particle, that is a convection rate constant of solution particle and a saturable transportation rate constant of solution particle. At secondary micro-level transportation, the results show convection as sum of advection and saturable transportation. The kinetics of dissolution of efavirenz in the solution space is postulated. Relatively, a good level of kinetics of dissolution is projected in the concentration region 0 − 32.82 μ*g/ml*.

## 1. Introduction

Efavirenz is an antiviral drug that has low solubility and high permeability (*pK_a_* = 10.2 and log *P* = 5.4) (Pinto et al., 2014). Efavirenz has been recommended as the preferred option for a non-nucleoside reverse transcriptase inhibitor in optimized first-line antiretroviral regimens (WHO, 2012; Ford et al., 2014). Efavirenz is a drug that exhibit low and variable oral bioavailability. Relationships of dissolution to bioavailability has been suggested (Dokoumetzidis and Macheras, 2006).

Modeling transportation has been mainly done using location and time space variables. The use of partial differential equations continues to be used in modeling diffusion patterns. Ogata and Banks (1961) derived the analytical solution to the advection-dispersion equation. Stochastic differential equations are also in use in modeling diffusion patterns, this has had extensions in areas of mathematics that include population dynamics and financial mathematics (Øksendal, 2000).

In the transportation of a drug there were inferences on the different possible transportation modes acting on the drug in the volumetric space. A solution particle in this work, is a zero sum movement entity for any given concentration consisting of movements due to concentration and the system's environment. It has equal pro-solvation and anti-solvation movement entities at any given concentration. Furthermore, it is the smallest particle of a solution. In this work mathematical models with biological implications are suggested. There are new parameters developed for the movement rates of solution particle. Furthermore, a proposition of an identification of the advection rate of solution particle parameter is given.

The Hill's equation was introduced by A. V. Hill to describe the relationship between oxygen tension and the saturation of hemoglobin a static effect (Goutelle et al., 2008). In this work it is used to describe saturation dissolution transportation in relation to concentrations of efavirenz. Additionally, the Ricker's model is used to model convection transportation rate of solution particle. This model has had applications in fishery, biological sciences, and population modeling in the description of “growth” rates in conditions where there is mortality and recruitment (Paz and Larraneta, 1992; Nelis, 2012; Wieland and Siegstad, 2012). When concentration was increasing in the volumetric space, the collective aggregated movement (convection) was expected to initially increase and then decrease. This was postulated to be mainly so, because of the finite volumetric space, elimination and uptake of the drug, amongst other factors that could potentially affect the convection rate as concentration increased.

The Michaelis-Menten equation is a special example of the Hill's equation and describes the relationship between the velocity of the reaction (dynamic effect) and the concentration of the substrate (Goutelle et al., 2008). It also describes non-linear saturation relationships. An energy dependent system transportation rate of solution particle is modeled by the Michaelis-Menten equation. The energy independent transportation rate of solution particle is modeled by the “inverse” Michaelis-Menten equation. This includes all forms of possible passive transportation rates of solution particle in the movement of a drug (efavirenz) in the volumetric space (Volume of Distribution) (Peck et al., 2008). The Michaelis-Menten equation is used because of saturation that was expected in transportation. As concentration increased in the systemic circulation there was an expected reduction in the rate due to dependency on concentration gradient for the passive transportation rate of solution particle.

The work made use of properties at the point associated with full absorption to help infer the possible transportation of the drug efavirenz for differing efavirenz plasma concentration with the aid of mathematical models. This work was motivated by the notion of attempting to find models with physiological rationale in the transportation of the drug efavirenz (Ette and Williams, 2007). Understanding transportation could also possibly help in explaining mechanisms that contribute to CNS side effects in the use of drugs (Nigam, 2015).

## 2. Materials and methods

Simulations were developed from 61 patients who had been on efavirenz containing HAART, this work made use of model 2b(i), Equations (1.4) and (1.10) in Nemaura (2014). The R Statistical package was used to further develop models in this work. There is use of the non-linear regression models in curve fitting. Furthermore, the following models were used, Ricker's Model, Michealis-Menten equation and the Hill's equation.

## 3. Results

An estimation of the behavior of the differing concentrations in the volumetric space was made at the point associated with full absorption. The point associated with full absorption was observed to be important and unique where *x_u_*(*t*) ≈ *x*(*t*) since the $\frac{\text{uptake-volume}(V)}{\text{volume of distribution}(V_{d})}\approx 1(x_{u}(t)=\frac{A}{V(1-k_{e})}(e^{-k_{e}t}-e^{-t})$ was taken as a function that defined full mass transfer of the drug, *A* was the absolute bioavailability, *k_e_* elimination rate constant and *V* = 35.56*L* was the uptake-volume (transportation volume) associated with full absorption, and *x*(*t*) was concentration at time *t*). A proposition on an equilibrium state in transportation was made at this point and captured by the transport equation (Equation 1.10) in Nemaura (2014), which could be written as

(1) $$k_{e}=-\frac{1}{\gamma }\left(\frac{D}{V}-\frac{A}{V}\right)$$

where *D* = 600 *mg* was the dose given. The factor −$\frac{1}{\gamma }$ represents transport rate into the opposing system. It was identified as the movement rate generated by the bulk movement of transportation volume containing efavirenz projected, and with the units $\frac{ml}{\mu g.h}$. The left side of Equation (1) represents a system which is only related to what had presumably reached the systemic circulation. The right side represents the complement. An assumption of equality of this rate to the system that empties into the systemic circulation at the point of full absorption was made. This was because of zero net flow of the drug in both systems that is the systemic system (that assimilated *A*) and its complement (that assimilated *D* − *A*). This point was regarded as an equilibrium point with respect to net flow.

### 3.1. Advection rate, convection rate, and *E_D_* and *E_I_* transportation rates

The movement rates of solution particle in this section describe the primary micro-level transportation system. The volumetric system was assumed to be homogeneous and transportation to be uniform for similar concentrations across individuals. This was due to possibilities of overlapping in substrate specificities among drug transporters and compensatory up-regulation of other drug transporters as a result of loss of the other (Nigam, 2015). Additionally, an assumption of instantaneous spread in the volumetric space was made. An assumption that advection at a macro-level as described above equals one at a micro-level thus, the advection rate of solution particle (inherent solution particle movement due to *anti-solvation/anti-dissolution* movement) was also given by −$\frac{1}{\gamma }$ and the following highly statistically significant non-linear regression fit between *x*(*x* ∈ (0, 15.5]) and *y* was obtained as (Equation 2 and Table 1),

(2) $$y=-\frac{1}{\gamma }=\underset{\text{I}}{\underset{︸}{gxe^{-hx}}}+\overset{\text{II}}{\overset{︷}{\frac{nx}{b+x}}}+\underset{\text{III}}{\underset{︸}{m\;\left(\frac{p}{x}+1\right)}}$$

where

*n*−maximum energy dependent (*E_D_*) system transportation rate of solution particle,

*b*−concentration at which *E_D_* transportation rate of solution particle was half of *n*,

*m*−minimum energy independent (*E_I_*) transportation rate of solution particle,

*p*−concentration at which *E_I_* transportation rate of solution particle was twice of *m*,

*g*−residence rate of the convection rate of solution particle,

*h*−declining rate of the convection rate of solution particle with increasing concentration.

**Table 1:** **Parameter estimates in modeling movement rates in Equation (2)**.

**Parameters**	**Estimate**	**Std Error**	***t*-value**	***Pr*(> |*t*|)**
*n*	−0.0561	0.0057	−9.764	5.18× 10^−10^
*b*	7.4315	0.6922	10.737	7.49×10^−11^
*g*	0.0089	0.0003	32.289	< 2 × 10^−16^
*h*	0.0598	0.0015	39.930	< 2 × 10^−16^
*p*	0.8808	0.0874	10.076	2.75×10^−10^
*m*	0.0034	0.0002	14.010	2.43×10^−13^

In Equation (2), part I− modeled convection rate of solution particle (inherent solution particle movement due to *pro-solvation* movement), part II− modeled the energy dependent system transport rate of solution particle, and part III− modeled passive transportation rate of solution particle.

As *x* → ∞ (as *x* gets large), II → *n*, III → *m*, and I → 0. For large values of concentration, the convection rate of solution particle was projected to be low in the volumetric space.

**Figure 1:**
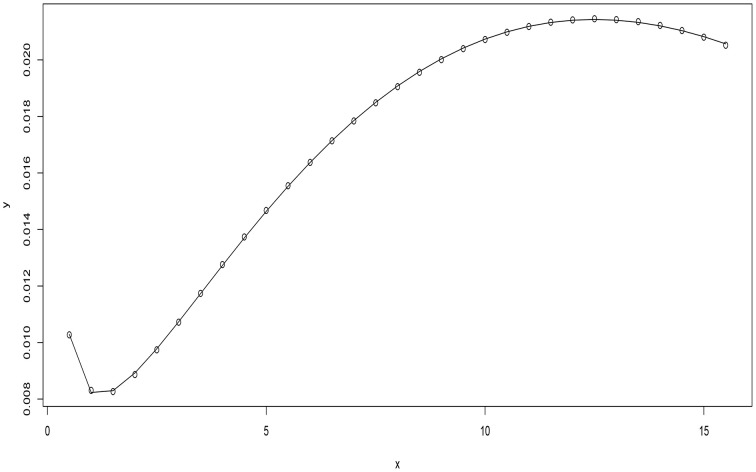
**The model fit of advection rate *y* against concentration *x***.

There were two turning points (Figure 1) one with the least advection rate of 0.0082 $\frac{ml}{\mu g.h}$ (minimum)at a concentration of 1.44 μ*g/ml*. The maximum advection rate was observed at concentration of 12.5 μ*g/ml*. At $\bar{x}$ = 0.97 μ*g/ml* the passive transport rate equated to the energy dependent system transportation rate. The equations that described the dominance of either energy dependent or energy independent system transportation were considered.

(3) $$\{\begin{array}{l} {\text{VT}}_{\text{II}}(x)>{\text{VT}}_{\text{III}}(x),\;\;x>0.97\;\mu g/ml\;\;\;\;\;\;\;\;\;\;\text{case}(i)\\ {\text{VT}}_{\text{II}}(\bar{x})={\text{VT}}_{\text{III}}(\bar{x}),\;\;x=\bar{x}=0.97\;\mu g/ml\;\;\;\;\text{case}(ii)\\ {\text{VT}}_{\text{II}}(x)<{\text{VT}}_{\text{III}}(x),\;\;x<0.97\;\mu g/ml\;\;\;\;\;\;\;\;\;\;\text{case}(iii) \end{array}$$

where VT_II_, and VT_III_ were absolute values of II and III in Equation (2). Considering Equation(s) (3) above it was deduced that for case (*i*) energy dependent system transportation rate of solution particle was dominating passive transportation since the magnitude of part II was greater than the magnitude of part III. The third case highlighted conditions where passive transportation rate of solution particle was dominating energy dependent system transportation rate of solution particle.

### 3.2. Transportation/movement rate constants and kinetics of dissolution of solution particle

Movement rate constants of solution particle are secondary micro-level transportation measures. There was consideration of the relationship between the proposed advection rate constant of solution particle, convection rate constant of solution particle and saturable transportation rate constant of solution particle to the concentration of efavirenz in the volumetric system. The advection rate constant of solution particle (*a_r_*) is defined as the product of advection rate of solution particle and relative uptake at the point of full absorption and is given by −$\frac{A}{\gamma V}$. The following highly statistically significant non-linear regression fit between *x*(*x* ∈ (0, 15.5]) and *a_r_*(*x*) was obtained (Equation 4 and Table 2)

(4) $$a_{r}:=-\frac{A}{\gamma V}=\underset{\text{q}}{\underset{︸}{\zeta xe^{-\lambda x}}}+\overset{\text{s}}{\overset{︷}{\frac{\alpha x^{\omega }}{\eta^{\omega }+x^{\omega }}}}$$

where

ζ−residence rate of the convection rate constant of solution particle,

λ−declining rate of the convection rate constant of solution particle with increasing concentration,

α−maximum saturable transportation rate constant of solution particle,

ω−Hill's coefficient and

η−concentration at which the saturable transportation rate constant of solution particle was half of α.

**Table 2:** **Parameter estimates in modeling movement rate constants of solution particle for Equation (4)**.

**Parameters**	**Estimate**	**Std Error**	***t*-value**	***Pr*(> |*t*|)**
ζ	0.1440	0.0040	35.69	< 2 × 10^−16^
λ	0.0596	0.0007	79.89	< 2 × 10^−16^
α	−0.7427	0.0555	−13.38	3.59 × 10^−13^
ω	1.1477	0.0123	92.98	<2 × 10^−16^
κ = η^ω^	6.071	0.2638	23.02	< 2 × 10^−16^

Considering Equation (4), q modeled convection rate constant of solution particle and s modeled saturable transportation rate constant of solution particle. Furthermore, the following were defined, q : = *c_r_*(*x*) and s : = −*st_r_*(*x*) where *c_r_*(*x*) was the convection rate constant of solution particle and *st_r_*(*x*) was the saturable rate constant of solution particle.

**Figure 2:**
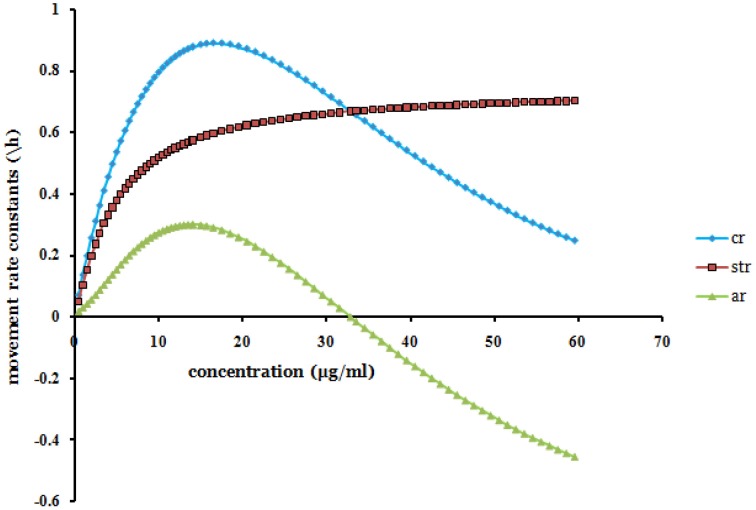
**The projected points (produced from Equation 4) of efavirenz plasma concentration (*x*) in the range 0 − 60 μ*g/ml* vs. rate constants of solution particle [advection rate constant of solution particle (*a_r_*), convection rate constant of solution particle (*c_r_*), and saturable transportation rate constant of solution particle (absolute (*st_r_*))]**.

Since α was found being negative (Table 2 and Equation 4) it was noted that *a_r_* + *st_r_* = *c_r_*. As *x* → ∞ (as *x* gets large), *a_r_*(*x*) → −*st_r_*(*x*) [and *c_r_*(*x*) → 0]. The movement rate constants of solution particle are bounded. The model (Equation 4 and Figure 2) thus showed that for large values of *x*, increasing efavirenz concentration decreases the convection rate constant of solution particle. Additionally, with increasing advection shows similar characterization to saturable transportation but in the opposite direction. This results in the convection approaching zero, thus implying less movement of the drug in the volumetric space at relatively high concentrations.

The saturable transportation rate constant of solution particle was shown to dominate the advection rate constant of solution particle (Figure 2). Extrapolations (Figure 2) based on the Equation (4) which were produced for the investigated range of 0 − 15.5 μ*g/ml* showed possibilities of negative advection rate constant of solution particle after *x_A_d__* = 32.82 μ*g/ml*. The extrapolation is observed when there was extension for all the values of concentration. Beyond this concentration value *x_A_d__*, efavirenz is postulated to have increasingly poor kinetic solubility. Kinetic solubility of solution particle, is the equilibrium movement solubility entity that constitute advection, saturation, and convection movements, of the dissolved or precipitated solution particle at secondary micro-level transportation for a given concentration and in a constant homogenous solution systems environment.

The following phases are postulated in relation to kinetics of dissolution and solubility of efavirenz. Initially, there are three important kinetics of dissolution boundary points defined.

1. The rest/zero kinetics of dissolution boundary point *x*_0_(*x*_0_ = 0) which is such that all movement entities are zero.
2. The central kinetics of dissolution boundary point *x_A_d__*(*x_A_d__* > 0) which is such that *a_r_*(*x_A_d__*) = 0 and *c_r_*(*x_A_d__*) = *st_r_*(*x_A_d__*) > 0.
3. The optimum kinetics of dissolution boundary point *x_C_*(*x_C_* > *x_A_d__* > 0) which is such that *c_r_*(*x_C_*) = 0 and −*a_r_*(*x_C_*) = *st_r_*(*x_C_*) > 0.

Three phases bounded by these kinetics of dissolution boundary points are proposed below

- **Kinetics of Dissolution Phase I** (*R_I_*): **Good Kinetics of Dissolution**
  *R_I_* = {*x* ∈ (0, *x_A_d__*): *a_r_*(*x*) > 0 and 0 < *st_r_*(*x*) < *c_r_*(*x*)}

1. The concentration *x* is in a very kinetic soluble region of *R_I_* if for ϵ_1_ > 0, *x* ∈ *N*_ϵ_1__(0).
2. The concentration *x* is in a freely kinetic soluble region of *R_I_* if for ϵ_1_, ϵ_2_ > 0, *x* ∈ [ϵ_1_, *x_A_d__* − ϵ_2_].
3. The concentration *x* is in a kinetic soluble region of *R_I_* if ϵ_2_ > 0, *x* ∈ *N*_ϵ_2__(*x_A_d__*).

- **Kinetics of Dissolution Phase II (*R_II_*): Poor Kinetics of Dissolution**
  *R_II_* = {*x* ∈ (*x_A_d__, x_C_*): *a_r_*(*x*) < 0 and 0 < *c_r_*(*x*) < *st_r_*(*x*)}

1. The concentration *x* is in a sparingly kinetic soluble region of *R_II_* if for ϵ_3_ > 0, *x* ∈ *N*_ϵ_3__(*x_A_d__*).
2. The concentration *x* is in a slightly kinetic soluble region of *R_II_* if for ϵ_3_, ϵ_4_ > 0, *x* ∈ [*x_A_d__* + ϵ_3_, *x_C_* − ϵ_4_].
3. The concentration *x* is in a very slightly kinetic soluble region of *R_II_* if for ϵ_4_ > 0, *x* ∈ *N*_ϵ_4__(*x_C_*).

- **Kinetics of Dissolution Phase III (*R_III_*): Undefined Kinetics of Dissolution(Insolubility)**
  *R_III_* = {*x* > *x_C_: c_r_*(*x*) = 0, *a_r_*(*x*) < 0 and *st_r_*(*x*) = −*a_r_*(*x*)}

At concentration *x* = *x_C_* the system has reached saturation and there is insolubility beyond this point. The volumetric solution space can “no longer” support solvation. The system has reached an “optimum dissolution capacity.” It should be noted that *st_r_*(*x*) = *st_r_*(*x_C_*), ∀*x* > *x_C_* (for every concentration above *x_C_*). Furthermore, the models show that a solution particle that is in the defined kinetics of dissolution region remains in this region as long as the pro-solvation movement of the solution particle can be accounted for however small. In otherwords, it continues to dissolve as concentration increases though with great difficulty. However, in reality when one can no longer account for pro-solvation at secondary micro-level of transportation (pro-solvation movement is very small/in the neighborhood of zero) an adoption of undefined dissolution kinetics (“no longer” dissolves) is made.

ϵ_1_, ϵ_2_, ϵ_3_, and ϵ_4_ are “small” radii of neighborhoods of kinetics of dissolution boundary points. The neighborhoods are pairwise disjoint, that is they describe different regions of kinetic solubility. Kinetic solubility is relative to concentration and solution system environment.

### 3.3. Acceleration rate constant of convection

The convection movement was tracked using the convection rate constants of solution particle projected using differing values of concentration for the patient who was projected as having the quickest flow of the drug (Patient P in Nemaura, 2014). In this section, the work only tracked the pro-solvation movement. This is done by finding the acceleration rate constant corresponding to convection rate constants for given concentrations in an individual. The convection rate constant of solution particle for the patient was estimated by the following model, since it mimicked the projected concentration curve,

(5) $$c_{r}(x(t))=\frac{j}{w-l}(e^{-lt}-e^{-wt})$$

where *t* ∈ [0, 24] (Table 3).

**Table 3:** **Parameter estimates in modeling convection rate constants of solution particle for Equation (5)**.

**Parameters**	**Estimate**	**Std Error**	***t*-value**	***Pr*(> |*t*|)**
*j*	0.332908	0.006517	51.08	< 2 × 10^−16^
*w*	0.340341	0.010495	32.43	< 2 × 10^−16^
*l*	0.032229	0.001198	26.89	< 2 × 10^−16^

The acceleration rate constant of solution particle corresponding to convection rate constant of solution particle of a given concentration was defined as the derivative of *c_r_*(*x*(*t*)) with respect to *t* (*t* ∈ (0, 24]), and was thus given by

(6) $$c_{r}^{\prime }(x(t))=\frac{j}{w-l}(we^{-wt}-le^{-lt})$$

**Figure 3:**
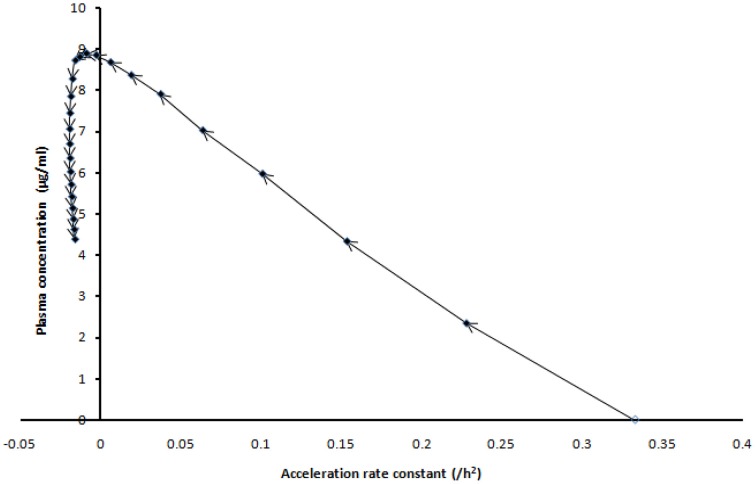
**The projected plasma concentration of patient *P* against the acceleration rate constant of solution particle at different hourly intervals (time-matched plot) for the duration of the dosing interval of efavirenz (24 h)**.

The acceleration was faster in the first few hours (Figure 3) as evidenced by the acceleration rate constant of solution particle and which was estimated to be 0.33/*h*^2^ in the neighborhood of 0 declining to −0.01596/*h*^2^ after 24 h. The negative acceleration was projected to be observed after 7 h. It should be noted that one can also track separate acceleration movements due to anti-solvation movements that is saturation and/or advection. The parameters introduced in this section could also be used to monitor Pharmacodynamic relations where transportation is a postulated to have an effect in patients.

## 4. Discussion

The models put forward in this work outlined several propositions. The energy dependent system transportation and energy independent system transportation are both involved in the transportation of the drug efavirenz in the volumetric space. The transportation attributed to convection can be modeled by the Ricker's model. A transportation mode that is independent of both advection and convection was found and was shown to be saturable at secondary micro-level transportation.

Using advection rate as the output measure, it was shown that convection rate of solution particle moves in the same direction as passive transportation rate of solution particle but is opposite to the energy dependent system transportation rate of solution particle. Considering convection rate of solution particle as the output measure for the primary micro-level transportation, it was shown that the movement rate is affected negatively by passive movement rate and positively by energy dependent rate and advection rate. Furthermore, an analysis of Equation (1) showed that the principal parameter (advection rate of solution particle) in modeling transportation can be modeled using the complementary system to the systemic circulation.

The equation modeling primary micro-level transportation suggested that for concentrations in the region *x* < 0.97 μ*g/ml*, passive transportation dominated the energy aided movement. In this same region, the system is in less control of efavirenz drug movement suggesting possibilities of ineffective therapy. The drug can go in and out of the system with relative ease and was inferred to be controlled mainly by concentration gradient. Moreover, Equation (1) showed that solution particle loses more movement to passive transportation at very low concentrations. Marzolini et al. (2001) found that 50% of patients with efavirenz levels of < 1 μ*g/ml* experienced virological failure. However, other researchers found no association between treatment failure and efavirenz concentrations below 1 μ*g/ml* (Borand et al., 2014). An experiment that track levels of efavirenz metabolites, efavirenz plasma concentration, efavirenz concentration at intracellular level, and the viral loads is required. This is in order to fully investigate what could potentially be happening so as to validate the results obtained by models of transportation that have been developed here. In addition, data on transporters and metabolizing enzymes associated with efavirenz metabolism and transportation and patient demographic data is also required.

Other studies have shown concentrations reaching close to and above 50 μ*g/ml* at steady state and investigations of transportation rate constants of concentrations up to 60 μ*g/ml* were thus considered (Nyakutira et al., 2008; Borand et al., 2014). Negative advection rate constant of solution particle reduces convection rate constant of solution particle. The sum of advection rate constant of solution particle and saturable rate constant of solution particle gives the convection rate constant of solution particle. The models gave evidence of possibility of advection being positive then negative with increasing concentration in the volumetric space. The maximum saturable rate constant of solution particle of efavirenz was found to be 0.7427/*h*. The secondary micro-level transportation system was used to infer on the kinetics of dissolution and solubility of efavirenz relative to concentration in the solution medium.

At primary micro-level transportation, energy independent transportation (*E_I_*) and convection describe pro-solvation movements while energy dependent transportation (*E_D_*) and advection are anti-solvation. Furthermore, at secondary level, the pro-solvation movement is convection while anti-solvation movement consists of saturation and advection. Additionally, this work also showed the importance of the mode of transportation and also concentration in the movement of the drug in the body and proposes links to solubility. During kinetics of dissolution phase I, the system supports only a completely dissolved state. In phase II, the system supports pro and anti-solvation states, thus we have dissolved and not dissolved elements of solution particle. However, in phase III the system no longer supports dissolution.

Dokoumetzidis and Macheras noted that mimicking simulation of the *in-vivo* hydrodynamic conditions at experiment level in attempting to explain dissolution is currently an obstacle (Dokoumetzidis and Macheras, 2006). However, this work attempts to give a mathematical description for the dissolution kinetics of efavirenz. The work tracks kinetic solubility by describing a solution particle's inside movement dynamics. Efavirenz has a small suggested therapeutic index (1−4 μ*g/ml*)^1^. This region falls in the expected good kinetics dissolution phase. Furthermore, the patients on efavirenz in the investigated sample have been classified to be in the good kinetics of dissolution region.

The investigated case of one patient's acceleration of convection, can be extended to the rest of the population in order to project the transportation behavior in this cohort. However, full profiles are required to ascertain the results projected here. This work intuitively looks at transportation in a volumetric medium with respect to time as compared to the methods in use to date which track movement with respect to area and time (Birger et al., 2014). It proposed models for the transportation modes. Furthermore, the work has found the existence of possible representation of transportation modes using mathematical models.

### Conflict of interest statement

The author declares that the research was conducted in the absence of any commercial or financial relationships that could be construed as a potential conflict of interest.

## Footnotes

^1^WHO. *Efavirenz Safety Review*. http://www.who.int/hiv/topics/treatment/efavirenz_safety_review.pdf.
